# Quality assurance of postharvest grapes against *Botrytis cinerea* by terbinafine

**DOI:** 10.1007/s13659-023-00389-w

**Published:** 2023-08-18

**Authors:** Yun Zhao, Qiong Jin, Zi-Jiao Wang, Xing-Yu Tao, Xiao-Dong Luo

**Affiliations:** 1 State Key Laboratory of Phytochemistry and Plant Resources in West China, Kunming Institute of Botany, Chinese Academy of Sciences, Kunming, 650201 People’s Republic of China; 2 Key Laboratory of Medicinal Chemistry for Natural Resource, Ministry of Education and Yunnan Province, Yunnan Characteristic Plant Extraction Laboratory, School of Chemical Science and Technology, Yunnan University, Kunming, 650500 People’s Republic of China; 3 University of Chinese Academy of Sciences, Beijing, 100049 People’s Republic of China

**Keywords:** Terbinafine, *Botrytis cinerea*, Antifungal activity, Cell membrane, Grape preservation

## Abstract

Worldwide, fruit is an indispensable treasure house of nutrition for human beings, occupying a vital position of human diet. Postharvest fruit storage requires efficient antifungal agents to control *Botrytis cinerea*, which is a vital postharvest disease affecting fruit and leading to enormous losses. However, with the enormous abuse of existing antifungal drugs, the problem of drug-resistant fungi is imminent, making the controlling diseases caused by pathogenic fungi even more challenging. Drug repurposing is an efficient alternative method, we evaluated a well-known antifungal chemical, terbinafine, against the agricultural pathogen, *B. cinerea *in vitro, as a result, terbinafine showed strong antifungal activity*.* Furthermore, the in vivo antifungal activity of terbinafine was evaluated, the results showed that terbinafine could reduce the decay area on grapes. Terbinafine could disrupt the cell membrane integrity, increase cell membrane permeability, and eventual cell death of *B. cinerea*. In addition, terbinafine reduced decay incidence, and weight loss and maintained the soluble solids, titratable acidity, ascorbic acid, total phenolic, and malondialdehyde content during the storage period of grapes. Overall, terbinafine could be an antifungal preservative for postharvest table grapes fresh-keeping.

**Graphical abstract:**

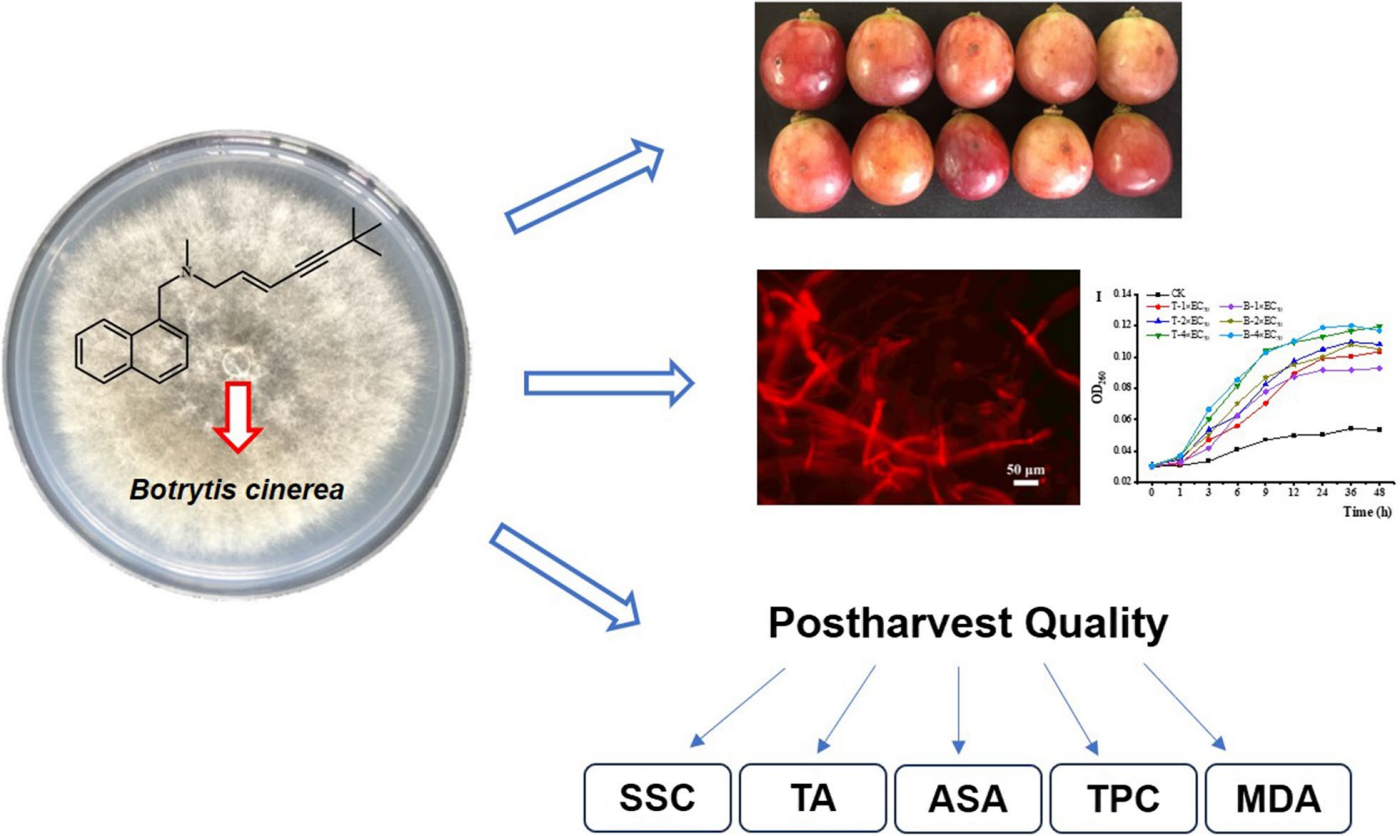

**Supplementary Information:**

The online version contains supplementary material available at 10.1007/s13659-023-00389-w.

## Introduction

Table grapes, as non-climacteric fruits with thin pericarp and succulent fruit tissue, are grown worldwide. Postharvest grapes are susceptible to infection by *Botrytis cinerea*, which leads to shorter storage time and huge economic losses [1]. Currently, there are various physical preservation technologies in the storage process, such as low temperature, air regulation and irradiation preservation, among which low-temperature preservation is widely used due to its low cost. However, the spores of *B. cinerea* can maintain strong pathogenic potential at low temperatures, and the occurrence of gray mold cannot be controlled by low-temperature preservation alone. Therefore, the main method of postharvest table grape preservation is the physical method combined with preservatives. Nevertheless, traditional fresh-keeping agents such as fludioxonil and carbendazim, are a serious threat to food safety due to their large dosage and high residue [2, 3], therefore, finding safe and effective fungicides is particularly urgent.

Sterol is an important component of the fungal cell membrane structure, which plays a vital role in ensuring the integrity of the membrane structure, the activity of membrane-bound enzymes, cell viability, and material transport [4]. Sterol biosynthesis inhibitors (SBI) are a class of systemic fungicides that inhibit the sterol biosynthesis of pathogens, and interfere with or block ergosterol biosynthesis by interacting with various enzymes in the sterol biosynthesis pathway, causing ergosterol deletion, and leading to structural abnormalities in the cell membrane and eventually achieving fungistatic and fungicidal effects [5]. Due to SBI have the characteristics of the wide antifungal spectrum, high efficacy, and strong selectivity, the development of SBIs has greatly promoted the progress of fungicides. Currently, SBI antifungal drugs in clinical use mainly include miconazole [6], and ketoconazole [7]. Meanwhile, SBI fungicides have not only made outstanding contributions in the field of medicine but also played an extremely important role in agricultural production. After spraying with imazalil, postharvest citrus can effectively avoid being infected by *Penicillium* spp. during the storage period [8].

**Fig. 1:**
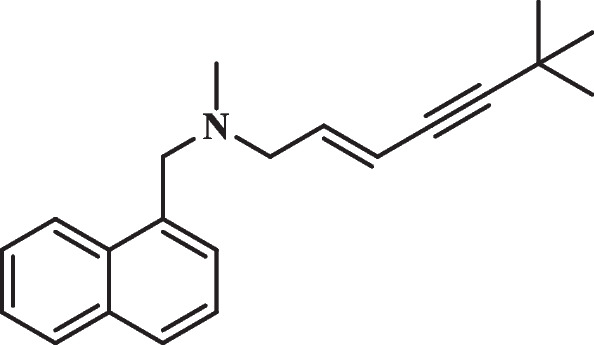
The structure of terbinafine

Terbinafine (Fig. 1), an allylamine chemical with a wide antifungal spectrum for human pathogens, can be synthesized chemically or found from the culture media of the *Streptomyces* sp., belongs to the SBI [9]. The potential mechanism against human pathogenic fungi of terbinafine specifically affects the process of ergosterol, inhibiting the enzyme squalene epoxidase, causing accumulation of squalene, and eventually leading to cell death [10]. However, the main cell membrane sterol component of humans is cholesterol, so human cells are much less sensitive to terbinafine than those of fungi. and the safety of terbinafine in humans has been verified in medical research, adults with oral terbinafine (500 mg·day^−1^) is well tolerated and safe [11]. Previous researches indicated that terbinafine can inhibit *Penicillium* spp., *Trichoderma* spp., *Acremonium* spp., and *Staphylococcus* spp. [12, 13] Therefore, based on the safety and broad-spectrum antifungal properties of terbinafine, it may have potential to be developed as an agricultural fungicide.

With the purpose of the drug repurposing strategy, we evaluated the in vitro and in vivo inhibitory effect of terbinafine against *B. cinerea* in table grapes. And the underlying antifungal mechanisms, such as plasma membrane integrity and cell membrane permeability were investigated. Furthermore, the present study is the first attempt to assess the effect of terbinafine on the postharvest fruit quality of table grapes during the storage period.

## Results

### Effect on mycelial growth of *B. cinerea*

**Fig. 2:**
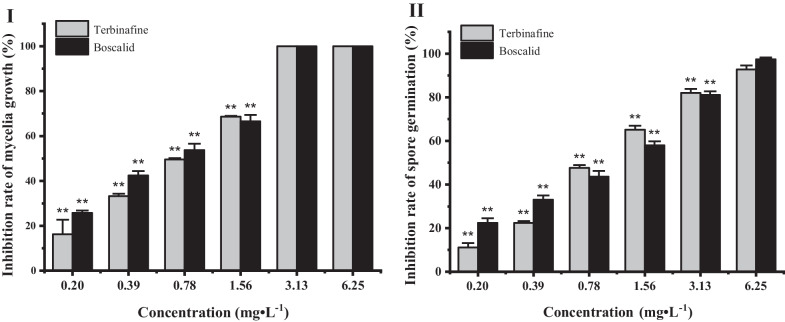
Effect of terbinafine hydrochloride and boscalid on mycelial growth **I** and spore germination **II** of *B. cinerea*. The concentration ranged from 0.20 to 6.25 mg·L^−1^. **P < 0.01 versus 6.25 mg·L^−1^ of terbinafine and boscalid, respectively

The inhibitory effect on mycelial growth of terbinafine hydrochloride and boscalid at different concentrations against *B. cinerea* is shown in Fig. 2I. The mycelial diameter decreased with increasing concentrations of drugs. At a concentration of 1.56 mg·L^−1^, the inhibition rates of terbinafine hydrochloride and boscalid were both more than 50%, and the antifungal activity of terbinafine hydrochloride was better than that of boscalid. The hyphal growth was completely inhibited after treatment with terbinafine hydrochloride and boscalid at 3.13 mg·L^−1^. The investigation reported a strong inhibitory effect of terbinafine against *B. cinerea*, the regression equation was y = 1.77x + 5.18, and the effective concentration for 50% of maximal growth inhibition (EC_50_) value was 0.80 mg·L^−1^ (Additional file 1: Table S1).

### Effect on spore germination of *B. cinerea*

Fungal conidia are the source of primary infection and reinfestation of plant diseases, which can be transmitted by air and rain, invade from stomata and mechanical damage, and produce diseases. As shown in Fig. 2II, chemicals can also inhibit the spore germination of *B. cinerea* in a dose-dependent manner. The results indicated that terbinafine could control disease occurrence at different developmental stages of fungi. However, the inhibitory effect of spore germination was weaker than that of mycelial growth, and the EC_50_ value of terbinafine hydrochloride against spore germination of *B. cinerea* was 0.95 mg·L^−1^ (Additional file 1: Table S2).

### Evaluation of plasma membrane integrity

**Fig. 3:**
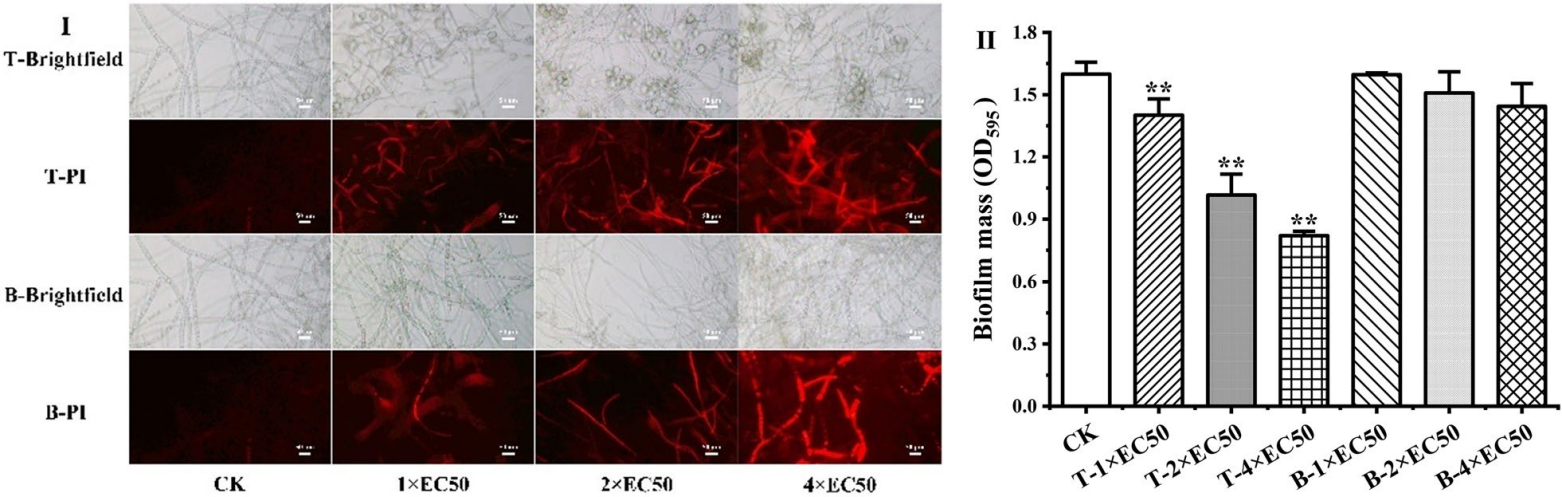
Effects of terbinafine hydrochloride (T) and boscalid (B) on plasma membrane integrity of *B. cinerea*. **I** The fungi were stained with propidium iodide (PI) and observed with a fluorescence microscope. The scale bar represents 50 *μ*m. **II** The biofilm mass was determined by the crystal violet (CV) assay (OD_595_). **P < 0.01 versus blank control

As shown in Fig. 3, the number of cells stained with propidium iodide (PI) increased with the increasing concentrations of drugs. After treatment with 4 × EC_50_ of terbinafine, a large number of red fluorescent signals were observed, indicating that terbinafine damaged the cell membrane integrity of the gray mold. The OD_595_ values showed that all compounds could inhibit cell membrane growth, and terbinafine was more effective than the positive control. The OD_595_ values were 1.02 and 0.82 after treatment with 2 × EC_50_ and 4 × EC_50_ of terbinafine, respectively, which were significantly different from that of blank control (1.60).

### Leakage of cytoplasmic contents

**Fig. 4:**
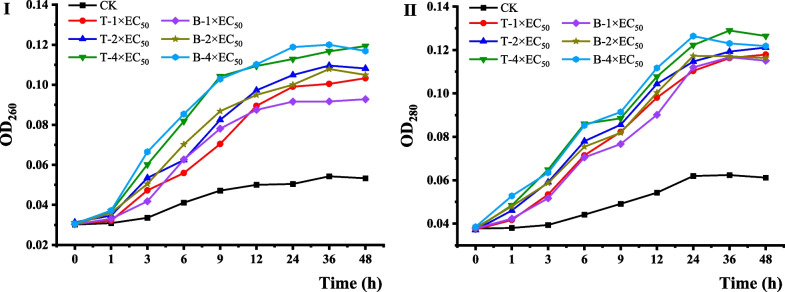
Leakage of cytoplasmic contents from *B. cinerea* treated with terbinafine hydrochloride (T) and boscalid (B) for 0, 1, 3, 6, 9, 12, 24, 36, and 48 h at concentrations of 1 × EC_50_, 2 × EC_50_, and 4 × EC_50_. **I** The nucleic acid content was expressed as the absorbance at 260 nm. **II** The protein content was expressed as the absorbance at 280 nm

The experimental results of PI staining and CV dying proved that the biofilm of *B. cinerea* was destroyed by terbinafine treatment. To disclose the potential mechanism of terbinafine against *B. cinerea*, therefore, we analyzed the leakage of cytoplasmic contents of *B. cinerea* treated with terbinafine. As shown in Fig. 4, nucleic acids and proteins both leaked from cells after treatment with terbinafine and boscalid. The values of OD_260_ and OD_280_ increased with increasing time.

### In vivo* inhibitory efficacy against B. cinerea*

**Fig. 5:**
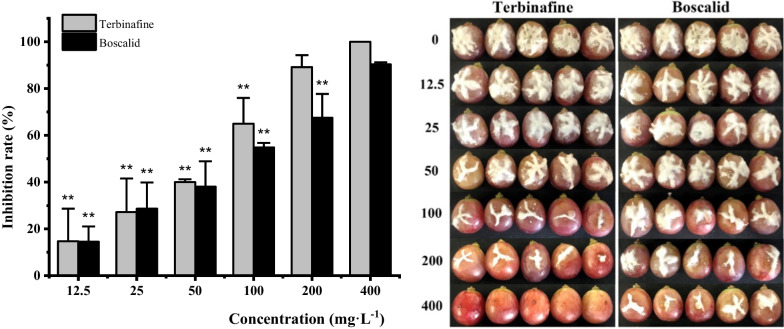
In vivo antifungal activities of terbinafine and boscalid against *B. cinerea* after 7 d of storage at 23 ± 1 ℃. The concentration ranged from 12.5 to 400 mg·L^−1^. **P < 0.01 versus 400 mg·L^−1^ of terbinafine and boscalid, respectively

This research indicated that terbinafine had strong antifungal activity against the mycelial growth and spore germination of *B. cinerea *in vitro. The in vivo inhibitory effect of terbinafine was similar to that in vitro, and the diameter and severity of the disease decreased with increasing treatment concentration (Fig. 5). At a concentration of 100 mg·L^−1^, the inhibition rates of terbinafine and boscalid were both more than 50%. Application of 400 mg·L^−1^ terbinafine prevented the development of gray mold decay, however, the inhibition rate of the positive control boscalid was only 90.3%. However, the effective concentration (EC_50_) value of terbinafine against *B. cinerea *in vivo was 56.4 mg·L^−1^, which was better than that of the well-known agricultural fungicide boscalid (70.9 mg·L^−1^) (Additional file 1: Table S3).

### Effects on postharvest quality of fresh table grapes

#### Decay incidence and weight loss

**Fig. 6:**
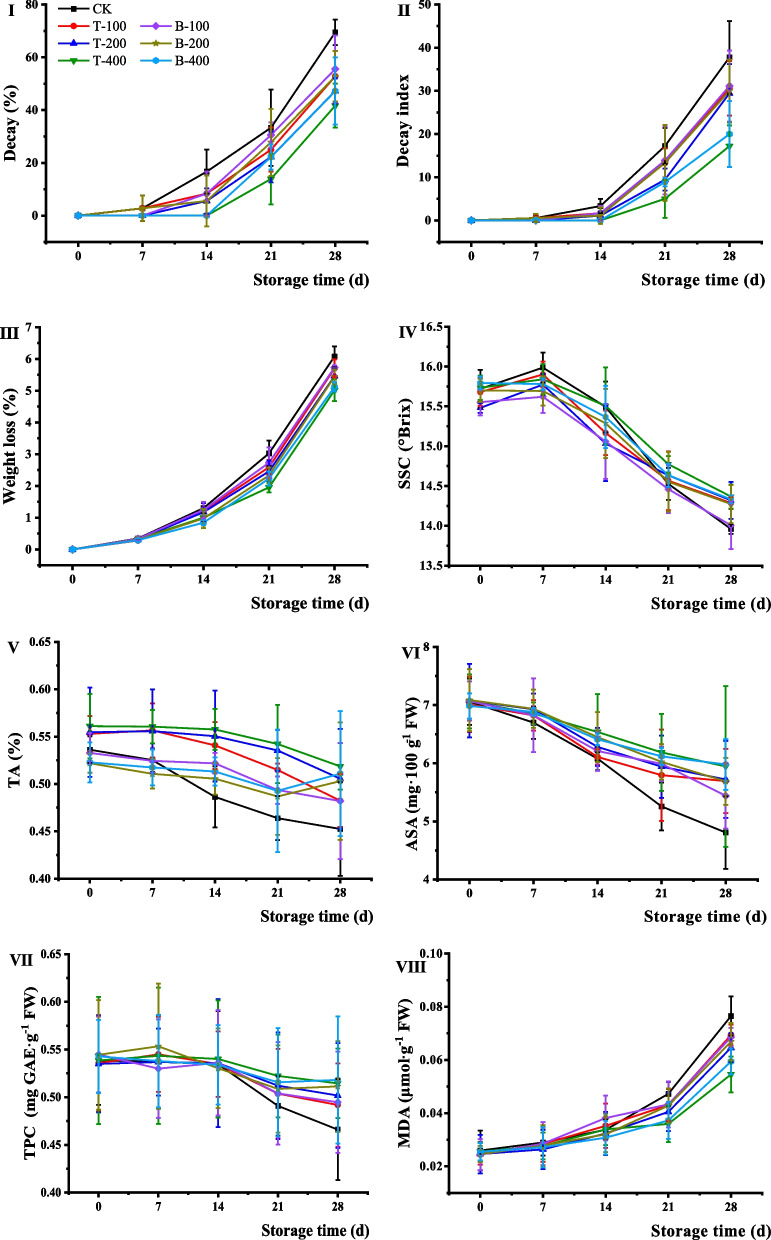
Quality of table grapes treated by terbinafine hydrochloride and boscalid on **I** decay percentage, **II** decay index, **III** weight loss, **IV** soluble solids content (SSC), **V** titratable acidity content (TA), **VI** ascorbic acid content (ASA), **VII** total phenolic content (TPC), and **VIII** malondialdehyde content (MDA) of grapes. T and B represented terbinafine hydrochloride and boscalid, respectively, and CK was the blank control

The effects of terbinafine and boscalid on the grape decay percentage and decay index are shown in Fig. 6. In treatments with or without drugs, the decay percentage and decay index increased with increasing storage time, and the increasing trend of the fruit rot rate in the late storage period was much greater than that in the early storage period. After 7 d of storage, the fruit of the blank control began to rot, and the sample exhibited a 69.4% decay incidence on the 28th day. The fruit decay percentage of the treatment group was significantly different from that of blank control, and the decay percentage was 47.2% with the 400 mg·L^−1^ terbinafine treatment on the 28th day. After being treated with terbinafine and boscalid on the 28th day of the storage period, the decay index was 17.2 and 20.0 respectively, which were lower than that of the blank control (37.8).

Changes in the weight loss of the control and treated grape fruit during storage are shown in Fig. 6III. The results revealed that the fruit weight decreased with increasing storage time, and the weight loss of the blank control was higher than that treated with terbinafine and boscalid. On the 14th day of storage, the weight loss of treatment groups and blank control was 0.85—1.30%. The grape weight loss rates were both less than 6% after treatment with terbinafine and boscalid at the 28th day of storage, of these, 400 mg·L^−1^ terbinafine was the most effective with a weight loss rate of only 5.02%, followed by 400 mg·L^−1^ boscalid (5.12%).

#### Soluble solids (SSC) and titratable acidity (TA) content

The total SSC content of table grapes showed a trend of increasing first and then decreasing (Fig. 6IV), and the treatment group could delay the decrease in SSC content in grapes during the whole storage process compared with blank control. Initially, the increased SSC content of grape fruit may be due to the hydrolysis of insoluble polysaccharides into simple sugars. As shown in Fig. 6V, the TA content decreased with increasing storage time, and the descending range in blank control was much greater than that in the treatment group.

#### Ascorbic acid (ASA) content and total phenolic content (TPC)

As shown in Fig. 6VI, the grape ASA content with or without drugs both decreased during the storage period, and the falling range of the blank control group was more than that of the treated group. The ASA contents were 6.99–7.09 mg·100 g^−1^FW at the beginning, and 6.08–6.45 mg·100 g^−1^ FW on the 14th day. Both terbinafine and boscalid can effectively inhibit vitamin C loss during the storage period, and 400 mg·L^−1^ terbinafine had the best inhibitory effect, with an ASA content of 5.98 mg·100 g^−1^ FW on the 28th day. The TPCs of the control and treatment groups both gradually increased until the 7th day and slowly decreased until the 28th day, and the TPC of the blank control was lower than that of the treatment group. After treatment with 400 mg·L^−1^ terbinafine, the TPC of grapes decreased by only 5.56% on the 28th day, while blank control decreased by 13.0% (Fig. 6VII).

#### Malondialdehyde (MDA) content

As shown in Fig. 6VIII, the MDA content increased slowly from 0 to the 14th day and increased rapidly after 14 days. After 28 days of incubation, the MDA content in grapes exposed to terbinafine at 400 mg·L^−1^ reached 0.05 *μ*mol·g^−1^ FW, which was significantly lower than that of the blank control (0.08 *μ*mol·g^−1^ FW).

## Discussion

Gray mold, caused by *B. cinerea*, is an aggressive postharvest disease and affecting fruit quality, causing fruit production to lose billions of dollars annually worldwide [14]. The result from this study indicated that treatment with terbinafine could effectively suppress *B. cinerea* disease in grape fruit. Although, many reports have demonstrated that terbinafine can efficiently inhibit *Penicillium* spp., *Trichoderma* spp., *Candida* spp. [12, 15], until now, there has few convincing evidence that terbinafine can control fruit postharvest disease in vivo. In the view of the application and safety of terbinafine in human health [16, 17], this study was evaluated the potential antifungal activity of terbinafine against *B. cinerea *in vivo, and expected to provide a potential beneficial antifungal approach for the control of postharvest diseases in fruit products.

In the present study, terbinafine effectively inhibited the mycelial growth, spore germination and germ tube elongation of *B. cinerea *in vitro. Complete inhibition of mycelial growth was observed at a concentration of 3.13 mg·L^−1^, same as the positive control. Previous study has shown that terbinafine hydrochloride has a significant inhibitory effect *B. cinerea* with the EC_50_ value of 0.11 mg·L^−1^ [18]. *B. cinerea* infection initiates when the spores tightly attach to the surface of grapes and produce germ tubes. Then the germ tubes can penetrate the fruit cuticle, and the pathogen establishes itself by producing highly differentiated hyphae, which results in gray mold disease. Terbinafine also inhibited the spore germination of *B. cinerea* at low concentrations (Fig. 2), and the EC_50_ value was only 0.95 mg·L^−1^. Fungicides with protective and curative effects can more effectively control the occurrence and spread of disease [19]. Compared with boscalid, terbinafine completely restrained the occurrence of gray mold decay at 400 mg·L^−1^, which showed direct evidence that terbinafine has the potential to be used to control *B. cinerea* during the storage period. Previous research synthesized five terbinafine ionic salts, and those compounds could effectively reduce infection diameters by *B. cinerea* on the cherry tomatoes at 20 mg/L [20].

The cell membrane plays an important role in the physiological functions of microorganisms, which can ensure the stability of the intracellular environment and regulate functions inside and outside the cell [21]. One of the main mechanisms of antifungal agents is the destruction of the cell membrane. A previous study revealed that miconazole, one of the SBIs, can strongly inhibit the biofilms of *Candida* spp. [22]. The inhibitory effect of terbinafine against *B. cinerea* may be attributed to the destruction of the membrane of the fungi, which is consistent with the result that terbinafine can destroy the biofilm growth of *C. albicans* [23]. In this study, intracellular leakage of nucleic acids and proteins of the pathogen during incubation was caused by treatment with terbinafine, which proved that the biofilm might be damaged or the biological function of the membrane system might be deranged after treatment. The amount of leakage increased with increasing time, which showed that the disintegration of cell membrane structures and the leakage of cellular contents was a slow process [24].

Due to the characteristics of thin skin, juiciness and fresh-eating of grapes, as well as the limitations of fresh-keeping technology, transportation, and storage conditions, grapes are overwhelmingly prone to rot during storage and transportation. Gray mold is one of the most common and destructive diseases of postharvest grapes [25], and the prevention and control of postharvest gray mold is the key to ensuring grape quality and economic benefits. After impregnating grapes with terbinafine solution, a barrier layer was formed on the fruit surface, which could effectively resist microbial infection from the external environment and reduce fruit spoilage. Fruit freshness and shelf life are closely related to water loss, which is due to transpiration and respiration, and the most direct manifestation of water loss is weight loss [26]. The observed results are probably related to a barrier created by terbinafine, which reduces water loss. The TA and TSS contents of grapes gradually decreased during the storage period, which is consistent with a previous study by Khaliq et al. [27] and Ehtesham et al. [28]. ASA, also known as vitamin C, has strong reducibility and is an important member of the antioxidant protection system of postharvest fruits, which can inhibit fruit degradation during the ripening process. The reduction in ASA content will lead to the accumulation of radicals, which will cause damage to cell tissues and accelerate fruit senescence [29]. Phenolic compounds are important secondary metabolites of fruits and vegetables and are closely related to the antioxidant capacity and quality of fruits and vegetables after harvest. The results indicated that terbinafine can effectively slow the loss of ASA and TPC content in grapes and improve the effect of grape preservation during the storage period. MDA is commonly used as an index of membrane lipid peroxidation to evaluate the degree of damage to the cell membrane system in fruits and vegetables. The higher the MDA content, the more serious the damage to the cell membrane structure. Fungal infection caused membrane lipid peroxidation during postharvest fruit storage, which increased the MDA content. Therefore, the change in MDA content was related to the degree of grape damage. Treatment with terbinafine could effectively inhibit the increase in MDA content, which better than that of boscalid, indicating that the fungal infection of grapes treated by terbinafine had been reduced. As shown in Fig. 6VIII, the MDA content increased with increasing storage time, which was consistent with previous research [30]; however, the increase in MDA content after treatment with terbinafine was slower than that of blank control.

## Materials and methods

### Materials

*B. cinerea* (Y-BC-1) was kindly provided by the State Key Laboratory for Conservation and Utilization of Bio-Resources in Kunming, Yunnan Province, P. R. China. Fresh table grapes (*Vitis vinifera*) were harvested from the local market, and fruit selection was based on uniformity in size, and color and without obvious injury. Terbinafine hydrochloride (purity 98%) was purchased from the Shanghai Acmec Biochemical Co., Ltd (Shanghai, China). Boscalid (purity 98%) was obtained from Shanghai Xianding Biotechnology Co., Ltd (Shanghai, China). All other chemical reagents were obtained from Macklin Reagents (Shanghai, China).

### Effect on mycelial growth of *B. cinerea*

The effect of terbinafine hydrochloride on mycelial growth against *B. cinerea* was determined by the mycelial growth rate method [31]. The terbinafine was dissolved by 0.5% dimethyl sulfoxide (DMSO). The mycelial plugs were cut from the edge of the 5-day-old colony and then placed in sterile potato dextrose agar (PDA) with terbinafine at final concentrations of 0.20, 0.39, 0.78, 1.56, 3.13, and 6.25 mg·L^−1^. PDA medium containing an equal concentration of boscalid, which is registered as a cure for gray mold, was considered a positive control, and 0.5% DMSO was the blank control. The Petri dishes were sealed with parafilm and incubated in the dark at 23 ± 1 ℃ for 5 d, and each concentration was performed three times. Mycelial radial growth was measured, and the antifungal activity was expressed as the effective concentration for 50% of the maximal effect (EC_50_). The relative inhibition of mycelial growth was determined using the following formula 1:

1 $${\text{Mycelial}}\,{\text{growth}}\;{\text{inhibition}}\,(\% ) = (C - T)/C \times 100$$

where *C* and *T* are the average diameters (mm) of *B. cinerea* mycelia in the blank control and the treatment, respectively. The linear regression equation (Y = a + bx) and the coefficient (R^2^) were estimated by SPSS 22.0 statistics software and the EC_50_ value was the logarithm value of x when Y = 5.

### Effect on spore germination of *B. cinerea*

The inhibitory effects of terbinafine against spore germination of *B. cinerea* were tested by direct contact assay with slight modifications [32]. First, the spore suspensions were harvested from seven-day-old cultures measured by a hemocytometer and adjusted to 1.0 × 10^5^ CFU·mL^−1^ with sterile potato dextrose broth (PDB). The conidial suspensions (190 *µ*l) were transferred to 96-well plates, and ten microliters of the stock solution of terbinafine hydrochloride was added to yield final concentrations of 0.20, 0.39, 0.78, 1.56, 3.13 and 6.25 mg·L^−1^. *B. cinerea* spores incubated in PDB with 0.5% DMSO were used as blank control, and boscalid at an equal concentration was used as a positive control. Then, the spores were incubated in the dark at 23 ± 1 ℃ for 6 h until the spores of the blank control germinated more than 90%, and the spore germination was determined microscopically (40 × 10) by counting 100 spores. Spores were considered germinated when the germ tube length was equal to or longer than the spore diameter. Three replicates were performed for each treatment. The regression equations and EC_50_ values were determined by the spore percentage inhibition and the chemical concentrations.

### Evaluation of plasma membrane integrity

The plasma membrane integrity was measured by using propidium iodide (PI) [33] and crystal violet (CV) [34]. Two hundred microliters of *B. cinerea* spore suspension (1.0 × 10^5^ CFU·mL^−1^) was incubated in sterilized 48-well plates at 23 ± 1 °C for 24 h. The supernatant was discarded and washed three times with phosphate-buffered saline (PBS, 0.01 mol·L^−1^, pH 7.2 ‒ 7.4). Then, *B. cinerea* spores were collected after treatment with sterile pure water and terbinafine or boscalid solution (1 × EC_50_, 2 × EC_50_, or 4 × EC_50_, EC_50_ means the value of the inhibition of 50% spore germination, the same below), respectively. Each treatment was repeated four times.

After incubating for another 24 h, the wells were washed three times with PBS, and 200 *μ*L of 0.1% CV was added to each well and incubated for 30 min. The cells were washed with PBS four times and solubilized with 200 *μ*L of 70% ethanol for 30 min, and, finally, 100 *μ*L of solution was transferred to a new 96-well plate and measured at 595 nm (Multiskan GO, Thermo, USA).

PI was dissolved in PBS at a final concentration of 10 mg·mL^−1^. Then stained samples with PI and incubated for 30 min at 23 ± 1 °C. Ultimately, washed them with PBS, and further observed under an Axio Vert A1 microscope (Zeiss, Germany) equipped with an X-Cite 120 Q (Lumen-Dynamics, Canada). PI cannot pass through living cell membranes, but it can penetrate into damaged cell membranes. Therefore, the higher the red fluorescence intensity, the more PI pass through damaged cell membranes and enters the cell [35].

### Detection of cellular leakage

The appropriate spore suspension (1.0 × 10^5^ CFU·mL^−1^) were cultured in sterile PDB, mycelia were collected and incubated in PBS containing terbinafine hydrochloride at different concentrations (1 × EC_50_, 2 × EC_50_, or 4 × EC_50_), and the supernatant was collected after 0, 1, 3, 6, 9, 12, 24, or 48 h. the mycelia were filtered through sterile filter paper, and the solutions were used to determine of the leakage of nucleic acids (OD_260_) and proteins (OD_280_) [36]. Four parallel replicates were set up in the experiment, and the positive control and blank control were boscalid and 0.5% DMSO, respectively.

### In vivo* inhibitory efficacy against B. cinerea*

Table grapes (*Vitis vinifera*) with no physical defects were sterilized with 75% ethanol, and then soaked with terbinafine solution (12.5, 25, 50, 100, 200, 400 mg·L^−1^) for 3 min and air-dried. A single artificial wound was made by a sterile nail at the equator in each fruit, and further inoculated with 10 *μ*L spore suspension (1.0 × 10^5^ CFU·mL^−1^) at each wound. Ten inoculated grapes were placed in sealed boxes and cultured at 23 ± 1 °C. An equal concentration of boscalid was a positive control and 0.5% DMSO was the blank control. Each treatment comprised three replicates. The lesion diameter was measured after 7 d. The proportion of rot area and the inhibition rate were calculated, and the antifungal efficiency was expressed by EC_50_ [37].

### Effects on postharvest quality of fresh table grapes

The postharvest grapes were washed with sterile water and respectively dipped in terbinafine hydrochloride solutions (100, 200, or 400 mg·L^−1^) for 3 min, and subsequently, air dried. Boscalid at an equal concentration was a positive control, and 0.5% DMSO was the blank control. Each treatment contained approximately 120 grapes, and all treated grapes were kept in plastic containers and stored at room temperature (25–30℃) with 85–90% relative humidity. The postharvest quality was measured by sampling at 0, 7, 14, 21, and 28 days.

#### Decay incidence

The decay incidence at the fruit surface was measured by the amount of rotten fruit and the intensity of microbial decay symptoms. The intensity of decay was reported by the following damage scale: 0 = no decay, 1 = one decay ≤ 2 mm in diameter, 2 = one decay ≤ 5 mm in diameter, 3 = several decays or 25% of berry surface contaminated, 4 = decay ≥ 26% of the fruit surface contaminated [38]. The decay index (DI) was calculated by formula 2:

2 $${\text{DI}} = \sum {(d \times f)/(N \times 5)}$$

where* d* is the degree of decay of the grape, *f* is its respective quantity, and *N* is the total number of fruits.

#### Weight loss

The weights of the treated and control fruit were recorded, and the weight loss was expressed as the reduction in weight as a percentage of total weight.

#### Soluble solids (SSC) and titratable acidity (TA) content

The soluble solids content was measured by a manual refractometer (LC-DR-32B, Shanghai Lichen Bangxi Instrument Technology Co., LTD, China) at room temperature, and the results were reported as °Brix. Fifty milliliters of distilled water were added to the fruit sample homogenate, and then the filtrate volume was determined. The TA was measured by titrating the filtrate against a standard solution of NaOH (0.1 M) using phenolphthalein as an indicator. The TA was expressed as% tartaric acid 100 g^−1^ fresh weight (FW) [39]. The experiment was performed in triplicate.

#### Ascorbic acid (ASA) content

The ascorbic acid content was determined based on the titration method [40]. A 5 g fruit sample was homogenized and mixed with 50 mL oxalic acid (2% w/v). Approximately 10 mL of filter liquor was titrated against 2, 6-dichlorophenol indophenol dye until the pink rose color was maintained for approximately 20 s. The ASA content was expressed as mg 100 g^−1^ ascorbic acid per fresh weight.

#### Total phenolic content (TPC)

The total phenolic content was measured as in a previous study with slight modifications [41]. Two grams of tissue was homogenized with 10 mL ethanol (80% v/v), extracted by ultrasonication at 40 ℃ for 60 min, and then centrifuged at 4000 × g for 20 min. Then, 0.1 mL of supernatant liquid was mixed with 0.3 mL Folin-Ciocalteu (0.5 mol·L^−1^) and 1.2 mL Na_2_CO_3_ (0.5 mol·L^−1^) and then filled with distilled water to 4 mL. After avoiding light for 1 h, the absorbance was read at 760 nm. Standard curves were made with different concentrations of gallic acid, and the results were expressed as mg·g^−1^ FW of gallic acid.

#### Malondialdehyde (MDA) content

The MDA content of table grapes was measured based on Wang et al. [42], with minor modifications. First, 1 g of tissue was homogenized with 3 mL of phosphate buffer solution (PB, 0.05 mol·L^−1^, pH 7.8), and then mixed with 5 mL of thiobarbituric acid (TBA, 0.5% w/v). The mixed liquor was heat-treated in a water bath for 10 min at 90℃, immediately cooled with ice, and then centrifuged at 3500 × g for 10 min. Finally, the supernatant absorbance was recorded at 450, 532, and 600 nm, and the MDA content was expressed as *μ*mol·g^−1^ FW.

### Statistical analysis

The SPSS software (Version 22.0, SPSS Inc., Chicago, IL, USA) was used to perform statistical analysis of the experimental data. Data were analyzed by one-way analysis of variance (ANOVA). Duncan’s multiple range test was applied to determine significant differences with the *p* < 0.01 level.

## Conclusion

Terbinafine showed strong antifungal activity of mycelial growth and spore germination against *B. cinerea *in vitro, and completely inhibited *B. cinerea* in artificially inoculated table grapes at 400 mg·L^−1^. In addition, the mode of action was attributed to the destruction of membrane structure and leakage of cellular contents. Furthermore, terbinafine treatment maintained the quality of the table grapes and extended their shelf life, and 400 mg·L^−1^ terbinafine was the most effective treatment in comparison to the positive control and blank control during the storage period by reducing the decay percentage, decay index, and weight loss, and maintaining the SSC, TA, ASA, TPC, and MDA contents of postharvest table grapes. In conclusion, terbinafine has the potential to be developed as an antifungal preservative for postharvest grape fresh-keeping.

### Supplementary Information

**Additional file 1.** Effective concentration (EC_50_) values of in vitro and in vivo antifungal activities against *B. cinerea* by the logarithm method.

## Data Availability

Data will be made available on request.
