# Supplementary material for: Quality assurance of postharvest grapes against Botrytis cinerea by terbinafine
Source: Nat Prod Bioprospect. 2023 Aug 18;13(1):25. doi: 10.1007/s13659-023-00389-w (PMC10439064; doi:10.1007/s13659-023-00389-w)
Supplement: Supplementary file 1 — Additional file 1. Effective concentration (EC50) values of in vitro and in vivo antifungal activities against B. cinerea by the logarithm method. [file 13659_2023_389_MOESM1_ESM.docx]

**Additional file 1**

**Quality assurance of postharvest grapes against *Botrytis cinerea* by terbinafine**

Yun Zhao^a,c^, Qiong Jin^a,c^, Zi-Jiao Wang^a,c^, Xing-Yu Tao^a,c^ and Xiao-Dong Luo^a,b,*^

*^a^ State Key Laboratory of Phytochemistry and Plant Resources in West China,* *Kunming Institute of Botany, Chinese Academy of Sciences, Kunming 650201, P. R. China*

*^b^ Key Laboratory of Medicinal Chemistry for Natural Resource, Ministry of Education and Yunnan Province, Yunnan Characteristic Plant Extraction Laboratory, School of Chemical Science and Technology, Yunnan University, Kunming 650500, P. R. China*

*^c^ University of Chinese Academy of Sciences, Beijing 100049, P. R. China*

__________________________________________________

*Corresponding author.

Email: xdluo@mail.kib.ac.cn;

Tel: +86-0871-65223177.


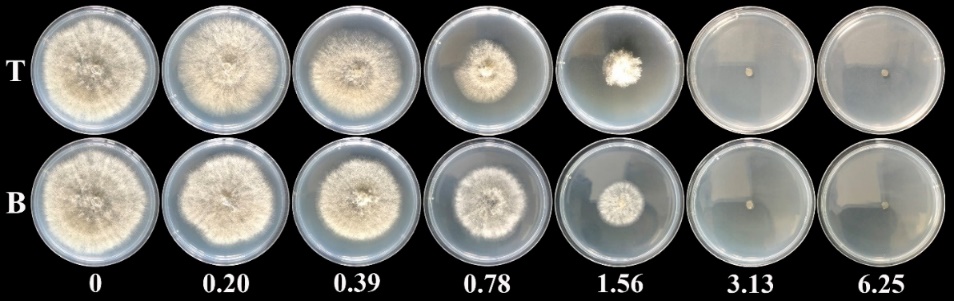


**Fig. S1.** Effect of terbinafine hydrochloride and boscalid on mycelial growth of *B. cinerea*. T and B represented terbinafine hydrochloride and boscalid, respectively. The concentration ranged from 0.20 to 6.25 mg/L.

**Table S1.** Effective concentration (EC_50_) values of terbinafine hydrochloride and boscalid against mycelial growth of *B. cinerea* after 5 d incubation at 23 ± 1℃ *in vitro*.

| Chemical | Regression equation | R^2^ | EC_50_  (mg/L) | 95% Confidence limits (mg/L) |
| --- | --- | --- | --- | --- |
| Terbinafine hydrochloride | y=1.77x+5.18 | 0.9892 | 0.80 | 0.58 ~ 1.08 |
| Boscalid | y=1.14x+5.19 | 0.9861 | 0.69 | 0.37 ~ 1.27 |


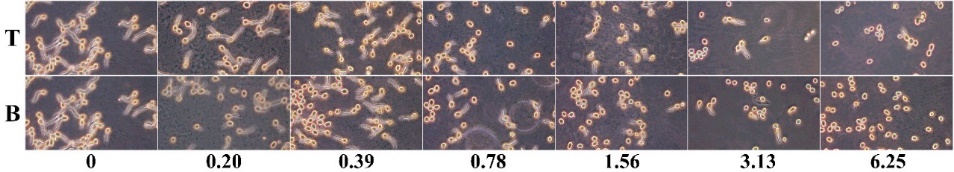


**Fig. S2**. Effect of terbinafine hydrochloride and boscalid on spore germination of *B. cinerea*. T and B represented terbinafine hydrochloride and boscalid, respectively. The concentration ranged from 0.20 to 6.25 mg/L.

**Table S2.** Effective concentration (EC_50_) values of terbinafine hydrochloride and boscalid against spore germination of *B. cinerea* after 6 h incubation at 23 ± 1℃ *in vitro*.

| Chemical | Regression equation | R^2^ | EC_50_  (mg/L) | 95% Confidence limits (mg/L) |
| --- | --- | --- | --- | --- |
| Terbinafine hydrochloride | y=1.79x+5.04 | 0.9973 | 0.95 | 0.82 ~ 1.10 |
| Boscalid | y=1.69x+5.21 | 0.9224 | 0.76 | 0.51 ~ 1.15 |

**Table S3.** Effective concentration (EC_50_) values of terbinafine hydrochloride and boscalid against *B. cinerea* after 7 d incubation at 23 ± 1℃ *in vivo*.

| Chemical | Regression equation | R^2^ | EC_50_  (mg/L) | 95% Confidence limits (mg/L) |
| --- | --- | --- | --- | --- |
| Terbinafine hydrochloride | y=2.37x+0.85 | 0.9934 | 56.4 | 50.3 ~ 63.1 |
| Boscalid | y=1.47x+2.27 | 0.9689 | 70.9 | 59.9 ~ 93.1 |
